# Notes and News

**Published:** 1906-03-10

**Authors:** 


### Notes and News.

At the annual Court of Governors of the Royal Berkshire Hospital, held at Reading last week, the resignation of Mr. F. C. C. Bennett, who had been Hon. Treasurer of the institution for five years, was announced. Colonel J. E. Broadbent was elected in his place.

During the time His Majesty's ship *Black Prince* was on view to the public, it was visited by no fewer than 21,000 persons, and the proceeds, amounting to £1,100 are to be divided between the Seamen's Hospital Society, and Poplar and West Ham Hospitals.

Mr. Winfrey has introduced into the House of Commons the Pharmacy Acts Amendment Bill, promoted by the Pharmaceutical Society with the view of remedying certain defects of the law concerning the sale of poisons. It is stated that he has obtained promises of support from a clear majority of members.

The latest contribution received at the Bank of England for King Edward's Hospital Fund for London includes £2,000 from Mr. Charles Morrison, £500 from Messrs. J. S. Morgan and Company, £105 from Mr. Howard Morley, £52 10s. from Messrs. Frank Bailey and Company, £50 from Lord Farquhar, and £50 from "W. J."

A reception will be given by the Lord Mayor and Lady Mayoress at the Mansion House on Friday, March 30, to the Presidents, Lady Presidents, Vice-Presidents, and Lady Vice-Presidents of the League of Mercy. Prince Alexander of Teck (President of the Kingston-Surbiton District of the League of Mercy) and Princess Alexander of Teck (Lady President of the Kingston-Surbiton and Epsom-Esher Districts) have graciously intimated their intention of being present.

The Goldsmiths' Company have made a grant of £10,000 to the Institute of Medical Science Fund, University of London, on the assumption that a site will be provided for the institute at South Kensington.

Surgeon-General James Pattison Walker, who died recently at Clacton-on-Sea, at the age of eighty-six, went out to India in 1845 in the Military Medical Service of the East India Company, and greatly distinguished himself during the Mutiny. Subsequently he filled the post of Professor of Hygiene in Calcutta, and Inspector-General of Hospitals in the North-West Provinces, retiring with the rank of Surgeon-General in 1877.

The death of Dr. Thomas Edmonton Charles, lately of the Indian Medical Service, an honorary physician to the King, and a deputy Surgeon-General, took place at Falmouth last week. Dr. Charles, who was in his seventy-second year, passed through the Indian Mutiny, and afterwards, in Calcutta, had for some years the largest professional connection of any English medical man. He also practised for seventeen years in Rome.

Lecturing at the Working Men's College on Saturday evening, Professor A. E. Shipley dwelt on the relation of flies to disease, and said that they had a nasty habit of dropping into milk and food. He had no doubt that they were to a great extent responsible for the prevalence of summer diarrhœa from which children suffered, and thus played a considerable part in bringing about the high infantile death-rate of which we heard so much in these days.

In the further report issued this week by the Royal Society of the Commission appointed by the Admiralty, the War Office, and the Civil Government of Malta, for the investigation of Malta fever, drastic preventive measures are recommended in order to hinder the disease from being transmitted by goats and mosquitoes to human beings. The oiling of stagnant water, with a view to the destruction of the larvæ of mosquitoes, is urged as a prime duty on the part of the sanitary authority.

The Executive Committee of the International Congress of Medicine have secured a considerable number of bedrooms for the use of visitors to Lisbon. A few of the rooms are single bedded, but the majority will contain several beds, and the price will be six, eight, and ten francs a night. There are also some boarding houses which will take in members at an inclusive charge of fifteen francs. Tickets for these lodgings will be obtainable at the Rocio station which is the terminus for passengers arriving at Lisbon by train. Visitors are required to pay for the whole eight days during which the Congress lasts. Accommodation will be reserved in strict accordance with the priority of application to Mr. Manoel Jose da Silva, Palacio Foz, Pracsa dos Restauradores, Lisbon. The hotels and restaurants as well as the buffet in the Congress buildings will solve the difficulty of obtaining meals.

